# Walk the path of cervical cancer elimination in Italy: current scenario and shared recommendations

**DOI:** 10.1093/eurpub/ckac131.131

**Published:** 2022-10-25

**Authors:** C de Waure, MT Riccardi, F D'Ambrosio, C Castagna, M Sapienza, R Millevolte, A Pellacchia, RP de Vincenzo, GE Calabrò

**Affiliations:** 1 Department of Medicine and Surgery, University of Perugia, Perugia, Italy; 2 Department of Life Sciences and Public Health, Università Cattolica del Sacro Cuore, Rome, Italy; 3 VIHTALI spin-off, Università Cattolica del Sacro Cuore, Rome, Italy; 4 Gynecologic Oncology Unit, Fondazione Policlinico Universitario A. Gemelli, Rome, Italy

## Abstract

**Issue/problem:**

In 2020, the World Health Organization (WHO) called for the elimination of cervical cancer. In order to get it, vaccination against Human Papillomavirus (HPV), screening of cervical cancer and treatment of high-grade cervical disease and cancer must be implemented at country level.

**Description of the problem:**

Italy has implemented HPV vaccination and cervical cancer screening for many years. Nevertheless, nationwide data show that both vaccination coverage and adherence to screening programs are unsatisfactory as compared to the WHO 90 and 70 targets, namely 90% of girls fully vaccinated by the age of 15 years and 70% of women screened with a high-performance test (i.e., HPV-DNA test) by age 35 and again by 45.

**Results:**

In order to address the progress of vaccination and screening at regional level in Italy, a project was conducted in 2021-2022 in order to collect data on relevant indicators and issues. In particular, information was collected on both coverage indicators (for both vaccination and screening) adherence (for screening) and history and characteristics of the vaccination offer (e.g., targets, gratuity) and of screening (e.g., presence of clinical pathways, type of tests used). Collected data were shared with a multidisciplinary panel of experts on HPV-related diseases to issue recommendations to foster the elimination of cervical cancer in Italy. For this purpose, a survey was also conducted to identify potential actions in respect to vaccination, screening and treatment.

**Lessons:**

A great heterogeneity across Italian regions was observed. The following actions were identified to implement vaccination, screening and treatment: educational campaigns, reminders and active calls for both vaccination and screening and more interoperability of data and definition of clinical pathway involving a multidisciplinary medical team for the proper management of all HPV-related diseases.

**Key messages:**

• Actions are requested at national level to achieve the goals set by the global strategy for cervical cancer elimination with respect to vaccination, screening and treatment.

• Actions identified to foster cervical cancer elimination in Italy includes educational campaigns, reminders and active calls, better interoperability of data and integrated medical team.

